# Efficacy of salvage therapies for advanced acral melanoma after anti-PD-1 monotherapy failure: a multicenter retrospective study of 108 Japanese patients

**DOI:** 10.3389/fmed.2023.1229937

**Published:** 2023-08-10

**Authors:** Tatsuhiko Mori, Kenjiro Namikawa, Naoya Yamazaki, Yukiko Kiniwa, Osamu Yamasaki, Shusuke Yoshikawa, Takashi Inozume, Hiroshi Kato, Yasuo Nakai, Satoshi Fukushima, Tatsuya Takenouchi, Takeo Maekawa, Shigeto Matsushita, Atsushi Otsuka, Motoo Nomura, Natsuki Baba, Taiki Isei, Shintaro Saito, Noriki Fujimoto, Ryo Tanaka, Takahide Kaneko, Yutaka Kuwatsuka, Taisuke Matsuya, Kotaro Nagase, Masazumi Onishi, Takehiro Onuma, Yasuhiro Nakamura

**Affiliations:** ^1^Department of Skin Oncology/Dermatology, Saitama Medical University International Medical Center, Saitama, Japan; ^2^Department of Dermatologic Oncology, National Cancer Center Hospital, Tokyo, Japan; ^3^Department of Dermatology, Shinshu University, Matsumoto, Japan; ^4^Department of Dermatology, Okayama University Graduate School of Medicine, Dentistry and Pharmaceutical Sciences, Okayama, Japan; ^5^Division of Dermatology, Shizuoka Cancer Center, Shizuoka, Japan; ^6^Department of Dermatology, Chiba University, Chiba, Japan; ^7^Department of Geriatric and Environmental Dermatology, Nagoya City University Graduate School of Medical Sciences, Nagoya, Japan; ^8^Department of Dermatology, Mie University, Mie, Japan; ^9^Department of Dermatology and Plastic Surgery, Faculty of Life Sciences, Kumamoto University, Kumamoto, Japan; ^10^Department of Dermatology, Niigata Cancer Center Hospital, Niigata, Japan; ^11^Department of Dermatology, Jichi Medical University, Tochigi, Japan; ^12^Department of Dermato-Oncology/Dermatology, National Hospital Organization Kagoshima Medical Center, Kagoshima, Japan; ^13^Department of Dermatology, Kyoto University, Kyoto, Japan; ^14^Department of Dermatology, Kindai University Hospital, Osaka, Japan; ^15^Department of Clinical Oncology, Kyoto University, Kyoto, Japan; ^16^Department of Dermatology, University of Fukui, Fukui, Japan; ^17^Department of Dermatologic Oncology, Osaka International Cancer Institute, Osaka, Japan; ^18^Department of Dermatology, Gunma University Graduate School of Medicine, Maebashi, Japan; ^19^Department of Dermatology, Shiga University of Medical Science, Otsu, Japan; ^20^Department of Dermatology, Kawasaki Medical School, Kurashiki, Japan; ^21^Department of Dermatology, Juntendo University Urayasu Hospital, Chiba, Japan; ^22^Department of Dermatology, Nagasaki University, Nagasaki, Japan; ^23^Department of Dermatology, Asahikawa Medical University, Asahikawa, Japan; ^24^Division of Dermatology, Department of Internal Medicine, Faculty of Medicine, Saga University, Saga, Japan; ^25^Department of Dermatology, Iwate Medical University, Iwate, Japan; ^26^Department of Dermatology, University of Yamanashi, Yamanashi, Japan

**Keywords:** melanoma, programmed cell death 1 receptor, immunotherapy, salvage therapy, nails

## Abstract

**Background:**

Anti-programmed cell death protein 1 (PD-1) monotherapy is one of the standard systemic therapies for advanced melanoma; however, the efficacy of salvage systemic therapies after PD-1 monotherapy failure (PD-1 MF), particularly in acral melanoma (AM), the main clinical melanoma type in Japanese patients, is unclear. This study aimed to investigate the efficacy of salvage systemic therapies in Japanese patients with AM after PD-1 MF.

**Patients and methods:**

The study included 108 patients with advanced AM (palm and sole, 72; nail apparatus, 36) who underwent salvage systemic therapy at 24 Japanese institutions. We mainly assessed the objective response rate (ORR), progression-free survival (PFS), and overall survival (OS).

**Results:**

Thirty-six (33%) patients received ipilimumab, 23 (21%) received nivolumab and ipilimumab (nivo/ipi), 10 (9%) received cytotoxic chemotherapy, 4 (4%) received BRAF and MEK inhibitors (BRAFi/MEKi), and the remaining 35 (32%) continued with PD-1 monotherapy after disease progression. The ORRs in the ipilimumab, nivo/ipi, cytotoxic chemotherapy, and BRAFi/MEKi groups were 8, 17, 0, and 100%, respectively. The nivo/ipi group showed the longest OS (median, 18.9 months); however, differences in ORR, PFS, and OS between the groups were insignificant. The OS in the nivo/ipi group was higher in the palm and sole groups than in the nail apparatus group (median: not reached vs. 8.7 months, *p* < 0.001). Cox multivariate analysis demonstrated that nail apparatus melanoma independently predicted unfavorable PFS and OS (*p* = 0.006 and 0.001). The total OS (from PD-1 monotherapy initiation to death/last follow-up) was insignificant between the groups.

**Conclusion:**

Nivo/ipi was not more effective than cytotoxic chemotherapy and ipilimumab after PD-1 MF in patients with advanced AM. The prognosis after PD-1 MF would be poorer for nail apparatus melanoma than for palm and sole melanoma.

## 1. Introduction

Malignant melanomas originate from melanocytes in the basal layer of the skin, mucosal epithelium, and uveal tract. The broad distribution of melanocytes throughout the human body leads to various clinical forms of melanoma, including nonacral cutaneous (NACM), acral (AM), mucosal, and uveal melanomas (1). AM, an uncommon melanoma subtype that arises from melanocytes in the volar skin and nail apparatus (2), accounts for 1–3% of all clinical melanoma forms in white-skinned populations in the United States and Europe (3, 4). Conversely, it is the most common clinical form among Asians (5–7), Latin Americans (4, 8), and Africans (3, 9), accounting for 40–71% of all melanomas. Furthermore, AM is often diagnosed at advanced stages, with approximately 60% of the patients being diagnosed with stage II or higher at the first examination (10). Therefore, it is critical to determine the clinical efficacy of systemic therapies for advanced AM. Additionally, AM reveals different mutation patterns of oncogenic drivers with NACM, including lower rates of *BRAF* (10–23%) and unstable *KIT* (3–29%) mutations (10, 11). The infrequency of these driver mutations decreases the probability of treating AM patients with targeted therapy, including BRAF and MEK inhibitor (BRAFi/MEKi) therapy and KIT inhibitor therapy. Therefore, there is an urgent need for effective immune checkpoint inhibitors (ICIs) and ICI-based therapies to treat advanced AM. Currently, anti-programmed cell death protein 1 antibody (PD-1) alone (nivolumab or pembrolizumab) or in combination with anti-cytotoxic T-cell antigen 4 antibody (CTLA-4) is the main treatment for advanced melanoma because of its favorable clinical efficacy, as shown in recent global phase III clinical trials (12, 13). However, these pivotal clinical trials had a small sample of patients with AM.

Phase II clinical trials in Japan (14, 15) have reported an approximate objective response rate (ORR) of 30% for nivolumab or pembrolizumab used to treat Japanese patients with advanced melanoma, including AM.

Meanwhile, ORR for toripalimab, which is another PD-1, was reported to be 14% in a Chinese prospective phase II study (POLARIS-01) involving 50 Chinese patients with AM (16). These ORRs were clearly lower than those reported in the global phase III clinical trials mentioned above. Additionally, recent retrospective studies have reported limited efficacy of PD-1 for advanced AM (17–25), particularly nail apparatus melanoma (NAM) (17, 20, 22). A recent retrospective study with a larger sample size also investigated the efficacy of PD-1 plus CTLA-4 for Japanese patients with advanced AM and demonstrated that the efficacy of PD-1 plus CTLA-4 was not superior to that of PD-1 monotherapy for palm and sole melanoma (PSM), although the efficacy of PD-1 plus CTLA-4 was potentially better than that of PD-1 alone for NAM (22). Because of the uncertainty regarding the superior clinical efficacy and the high incidence of severe adverse events compared to that with PD-1 monotherapy, PD-1 plus CTLA-4 is not necessarily the standard of care, and PD-1 monotherapy is still frequently used as the first-line treatment for advanced AM in Japan. Despite the need for salvage therapies after disease progression by PD-1 monotherapy (PD-1 monotherapy failure: PD-1 MF) in patients with AM in real-world practice, their detailed clinical efficacy remains unclear. Thus, this study aimed to investigate the clinical efficacy of various salvage therapies, including BRAFi/MEKi, cytotoxic chemotherapies, and ICIs, after PD-1 MF in patients with advanced AM.

## 2. Methods

### 2.1. Patients and study design

This multi-institutional (*n* = 24) retrospective observational study evaluated patients with advanced AM (unresectable stage III or IV) who initiated salvage therapies within 3 months of PD-1 MF or continued PD-1 after PD-1 MF (beyond progression [BP] use) between 2014 and 2020 in Japan. Patients who had received ipilimumab (ipi) before PD-1 monotherapy for an advanced-stage disease or had a history of prior adjuvant PD-1 monotherapy were excluded. The inclusion criteria were: age ≥ 18 years at PD-1 therapy initiation and a confirmed AM diagnosis. Staging followed the 8th American Joint Committee on Cancer (AJCC) staging system for cutaneous melanoma (26). Data (age, sex, clinical form, Eastern Cooperative Oncology Group performance status [ECOG-PS], AJCC stage, presence of *BRAF* mutations, baseline lactate dehydrogenase [LDH] level, number of metastatic organs at ICI initiation, and type of resistance to PD-1) were collected from the electronic medical charts. The PD-1 resistance types included innate resistance, defined as progressive or stable disease <6 months as the best response, and acquired resistance, defined as progressive disease after the initial complete or partial response or clinical benefit (stable disease for over 6 months), following the study by Pires da Silva et al. (27).

This study followed the STROBE guidelines (28). The Institutional Review Boards of the participating institutions approved this study (20–109), which was conducted following the Declaration of Helsinki. The requirement for informed consent was waived because of the retrospective nature of the study and use of anonymized data.

### 2.2. Efficacy assessment

The co-primary outcomes were ORR, progression-free survival (PFS), and overall survival (OS), defined from the date of salvage therapy initiation to progression, death, or last follow-up, respectively. The secondary outcomes were the salvage therapy disease control rate (DCR) and total OS (from PD-1 monotherapy initiation to death/last follow-up). Radiologic response and progression were assessed by board-certified radiologists/independent investigators at each institution following the Response Evaluation Criteria in Solid Tumors (Version 1.1) (29).

### 2.3. Statistical analyses

Baseline characteristics were compared using Fisher’s exact test for categorical variables and the Mann–Whitney *U* test and Steel–Dwass test for continuous variables. Survival (PFS, OS, and total OS) is expressed as medians and two-sided 95% confidence intervals (CIs). The survival curves were estimated using Kaplan–Meier method and compared using log-rank test. Cox proportional hazards analysis was used to identify independent predictors of PFS and OS after salvage therapy. Multivariate analysis was performed to account for potential confounding factors, including age, ECOG-PS, stage, LDH level, metastatic organ site, clinical form, PD-1 monotherapy resistance type, and salvage therapy type. Statistical analysis was conducted using JMP Pro, Version 16 (SAS Institute, Cary, NC, United States). Differences with *p* < 0.05 were considered statistically significant.

## 3. Results

### 3.1. Patients’ characteristics

We enrolled 108 patients with advanced AM (PSM, *n* = 72 [PSM group]; NAM, *n* = 36 [NAM group]) who developed progressive disease after first-line ICI treatment with PD-1 in our previous study (Table 1) (22). The median age was 72 (range, 34–92) years. There were 43 (40%) women and 65 (60%) men. Most (75%) patients had an ECOG-PS of 0. Patients with unresectable stages III, IV-M1a, IV-M1b, IV-M1c, and IV-M1d accounted for 23, 26, 21, 22, and 7%, respectively. Eight (7%) patients had brain metastases. Most (67%) patients had normal LDH levels and did not harbor *BRAF* mutations (89%). Innate and acquired resistance to PD-1 were observed in 69 and 31% of the patients, respectively.

**Table 1:** Patient baseline characteristics.

Baseline characteristics		No. of patients (%)					
		Total	BRAFi/MEKi	Chemotherapy	ipi	nivo/ipi	PD-1 BP
		*n* = 108	*n* = 4	*n* = 10	*n* = 36	*n* = 23	*n* = 35
Clinical form	PSM	72	4	4	21	16	27
	NAM	36	0	6	15	7	8
Median age (range)		72 (34–92)	63 (49–85)	73 (60–82)	69 (39–88)	69 (34–87)	78 (57–92)
Sex	Female	43 (40)	2 (50)	3 (30)	14 (39)	6 (26)	18 (51)
	Male	65 (60)	2 (50)	7 (70)	22 (61)	17 (74)	17 (49)
ECOG PS	0	81 (75)	3 (75)	9 (90)	25 (69)	18 (78)	26 (74)
	1	22 (20)	0	0	8 (22)	5 (22)	9 (26)
	≥2	5 (5)	1 (25)	1 (10)	3 (8)	0	0
BRAF mutation	Wild type	96 (89)	0	10 (100)	33 (92)	22 (96)	31 (89)
	Mutation	7 (6)	4 (100)	0	1 (3)	1 (4)	1 (3)
	Not investigated	5 (5)	0	0	2 (6)	0	3 (9)
Stage (AJCC-TNM 8th)	Unresectable stage III	25 (23)	1 (25)	1 (10)	7 (19)	8 (35)	8 (23)
	Stage IV (M1a)	28 (26)	2 (50)	4 (40)	6 (17)	3 (13)	13 (37)
	Stage IV (M1b)	23 (21)	0	2 (20)	8 (22)	8 (35)	5 (14)
	Stage IV (M1c)	24 (22)	0	3 (30)	13 (36)	2 (9)	6 (17)
	Stage IV (M1d)	8 (7)	1 (25)	0	2 (6)	2 (9)	3 (9)
LDH level	≤ULN	72 (67)	2 (50)	8 (80)	24 (67)	18 (78)	20 (57)
	>ULN	36 (33)	2 (50)	2 (20)	12 (33)	5 (22)	15 (43)
Metastatic organ sites	1	62 (57)	2 (50)	5 (50)	19 (53)	15 (65)	21 (60)
	2	17 (16)	1 (25)	1 (10)	6 (17)	4 (17)	5 (14)
	≥3	29 (27)	1 (25)	4 (40)	11 (31)	4 (17)	9 (26)
Brain metastasis	Absent	100 (93)	3 (75)	10 (100)	34 (94)	21 (91)	32 (91)
	Present	8 (7)	1 (25)	0	2 (6)	2 (9)	3 (9)
Type of resistance to PD-1 monotherapy	Innate	74 (69)	3 (75)	6 (60)	28 (78)	16 (66)	21 (60)
	Acquired	34 (31)	1 (25)	4 (40)	8 (22)	7 (30)	14 (40)

PD-1, anti-programmed cell death protein 1; BRAFi/MEKi, BRAF and MEK inhibitor; ipi, ipilimumab; nivo/ipi, nivolumab and ipilimumab; BP, beyond progression; PSM, palm and sole melanoma; NAM, nail apparatus melanoma; PS, performance status; LDH, lactate dehydrogenase.

Among the patients, 36 (33%) received ipi, 23 (21%) received nivolumab and ipilimumab (nivo/ipi), 10 (9%) received cytotoxic chemotherapy (9 dacarbazine and 1 temozolomide), 4 (4%) received BRAFi/MEKi, and the remaining 35 (32%) received PD-1 BP after PD-1 MF. Table 1 summarizes the detailed characteristics of patients in each treatment group.

Patients in the PD-1 BP group were numerically older while those in the BRAFi/MEKi group were numerically younger than those in the other groups. *BRAF* mutation was more frequently detected (100%) in the BRAFi/MEKi group than in the other groups. The proportion of patients whose LDH level was exceeding the upper limit of normal was numerically higher in the BRAFi/MEKi (50%) and PD-1 BP groups (43%).

### 3.2. ORR and DCR

The ORRs in the BRAFi/MEKi, cytotoxic chemotherapy, ipi, and nivo/ipi groups were 100, 0, 8, and 17%, respectively (Table 2). Although the ORR in the nivo/ipi group tended to be higher than that in the cytotoxic chemotherapy and ipi groups, the differences were insignificant (*p* = 0.40); the BRAFi/MEKi group was excluded from analysis due to its small size (*n* = 4). The respective DCRs were 100, 30, 28, and 39% (Table 2). The DCR in the nivo/ipi group was also insignificantly higher than that in the cytotoxic chemotherapy and ipi groups (*p* = 0.60).

**Table 2:** Overall response in each salvage therapy.

	No. of patients (%)				*P* *
	BRAFi/MEKi	Chemotherapy	ipi	nivo/ipi	
	*n* = 4	*n* = 10	*n* = 36	*n* = 23	
Complete response	1 (25)	0	0	1 (4)	
Partial response	3 (75)	0	3 (8)	3 (13)	
Stable disease	0	3 (30)	7 (19)	5 (22)	
Progressive disease	0	7 (70)	26 (72)	14 (61)	
Unable to determine	0	0	0	0	
Objective response	4 (100)	0	3 (8)	4 (17)	0.40
Disease control rate	4 (100)	3 (30)	10 (28)	9 (39)	0.60

BRAFi/MEKi, BRAF and MEK inhibitor; ipi, ipilimumab; nivo/ipi, nivolumab and ipilimumab.

* BRAFi/MEKi group was excluded from analysis due to its small size.

### 3.3. PFS, OS, and total OS

**Figure 1:**
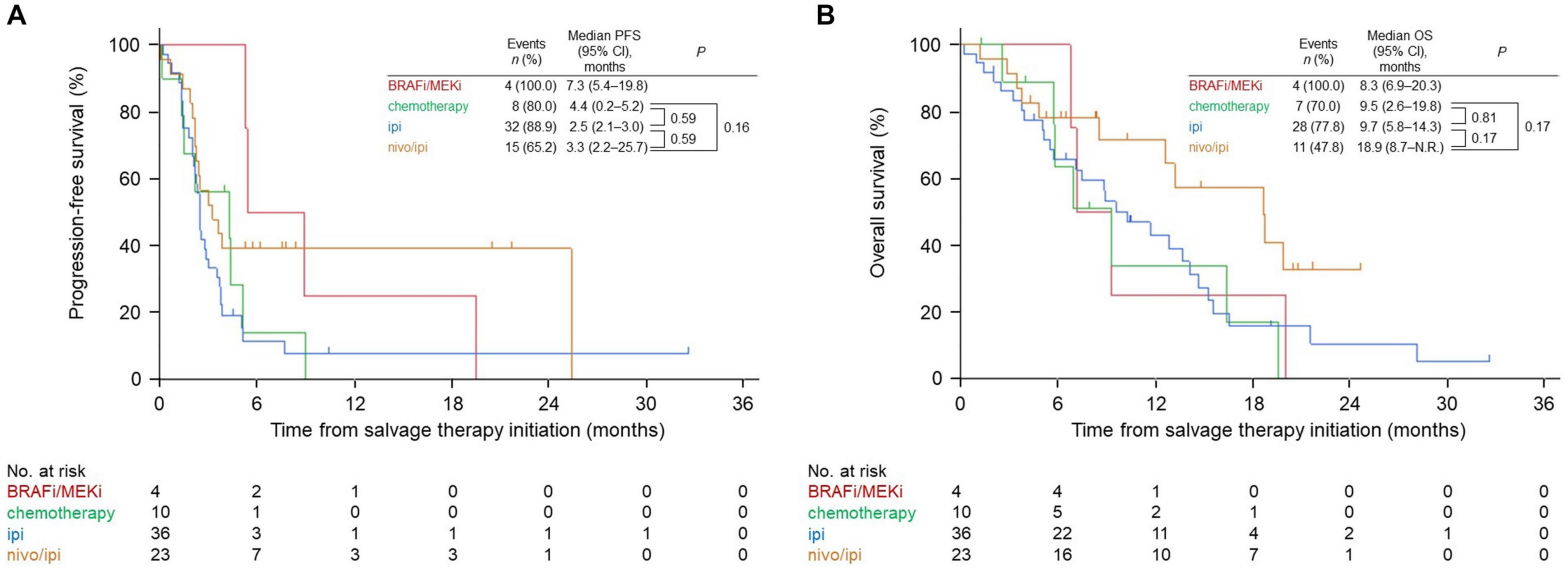
Kaplan–Meier survival curves for the salvage therapies. **(A)** Progression-free survival (PFS). **(B)** Overall survival (OS). BRAFi/MEKi, BRAF and MEK inhibitor; ipi, ipilimumab; nivo/ipi, nivolumab and ipilimumab; CI, confidence interval; N.R., not reached.

The median PFS in the nivo/ipi group (3.3 months [95% CI: 2.2–25.7]) differed insignificantly from that in the BRAFi/MEKi (7.3 months [95% CI: 5.4–19.8]), cytotoxic chemotherapy (4.4 months [95% CI: 0.2–5.2]; *p* = 0.16), and ipi (2.5 months [95% CI: 2.1–3.0]; *p* = 0.59; Figure 1A) groups. Likewise, the median OS in the nivo/ipi group (18.9 months [95% CI, 8.7–not reached]) was insignificantly longer than that in the BRAFi/MEKi (8.3 months [95% CI, 6.9–20.3]), cytotoxic chemotherapy (9.5 months [95% CI, 2.6–19.8]; *p* = 0.17), and ipi (9.7 months [95% CI, 5.8–14.3]; *p* = 0.17) (Figure 1B) groups, The BRAFi/MEKi group was excluded from analysis due to its small size (*n* = 4).

**Figure 2:**
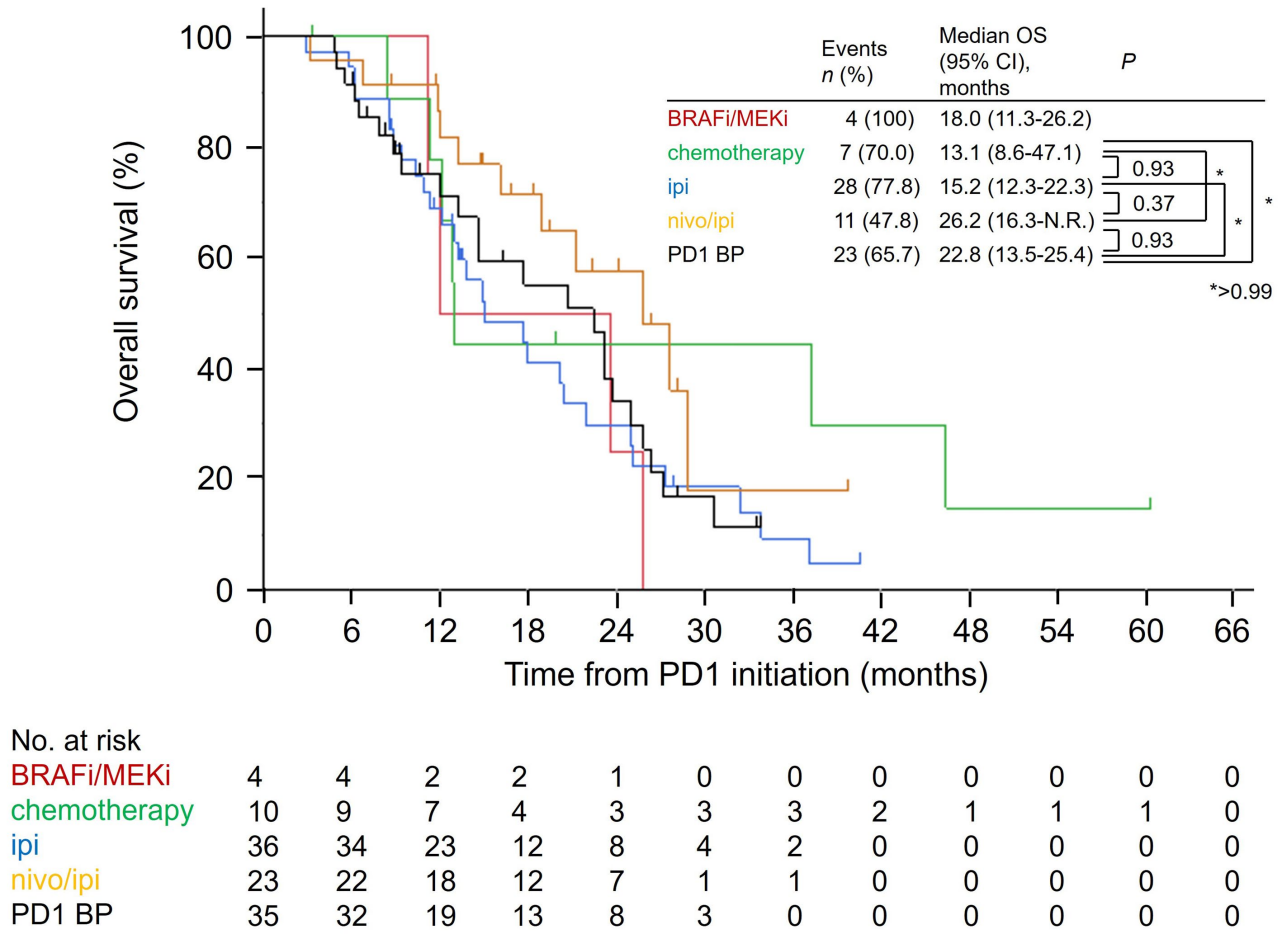
Kaplan–Meier overall survival curves for the salvage therapies from the PD-1 monotherapy initiation date. PD-1, anti-programmed cell death protein 1; BRAFi/MEKi, BRAF and MEK inhibitor; ipi, ipilimumab; nivo/ipi, nivolumab and ipilimumab; BP, beyond progression; OS, overall survival; CI, confidence interval; N.R., not reached.

We also evaluated the total OS in the salvage therapy groups. The median total OS in the nivo/ipi group (26.2 months [95% CI: 16.3–not reached]) was insignificantly higher than that in the other groups (cytotoxic chemotherapy: 13.1 months [95% CI: 8.6–47.1], *p* > 0.99; ipi: 15.2 months [95% CI: 12.3–22.3], *p* = 0.37; PD-1 BP: 22.8 months [95% CI: 13.5–25.4], *p* = 0.93; Figure 2).

### 3.4. Subgroup analysis in the nivo/ipi group

**Figure 3:**
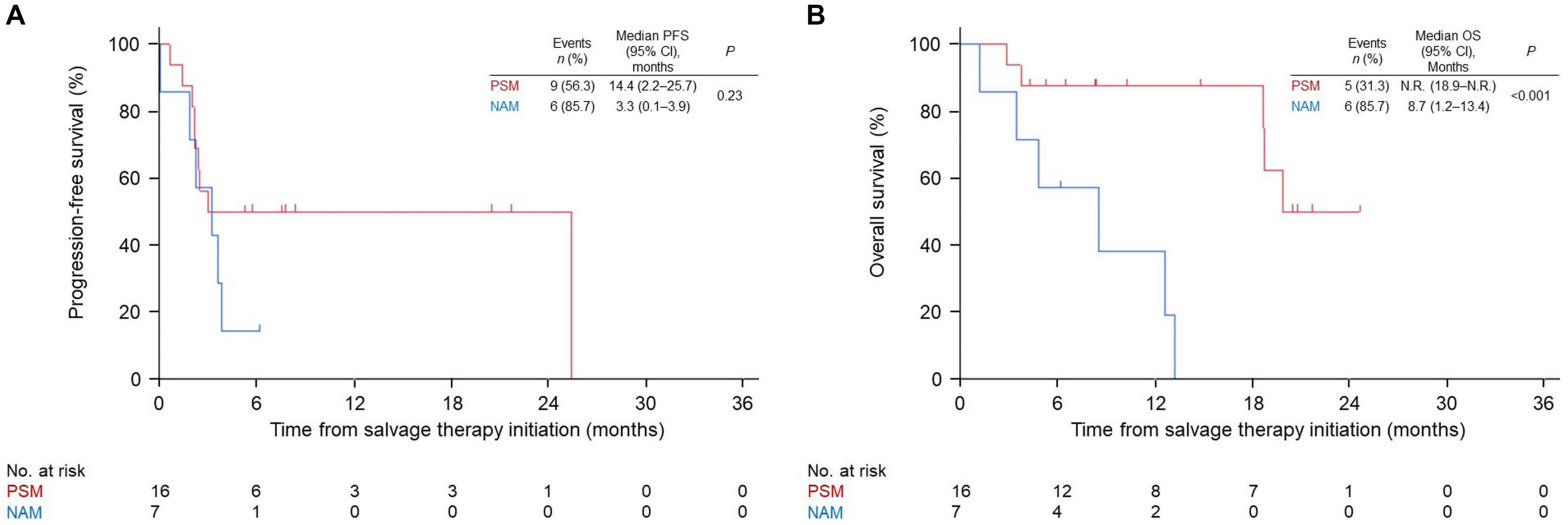
Kaplan–Meier survival curves for the palm and sole and nail apparatus melanoma subgroups in the nivolumab and ipilimumab combination group. **(A)** progression-free survival (PFS). **(B)** overall survival (OS). PSM, palm and sole melanoma; NAM, nail apparatus melanoma; CI, confidence interval; N.R., not reached.

Patients with AM in the nivo/ipi group were divided into PSM and NAM subgroups, and their ORR, PFS, and OS were compared. The two subgroups had similar baseline characteristics (Table 3). The ORR and PFS in the PSM subgroup were insignificantly higher than those in the NAM subgroup (ORR: 25 vs. 0%, *p* = 0.27; median PFS: 14.4 [95% CI: 2.2–25.7] vs. 3.3 months [95% CI: 0.1–3.9], *p* = 0.23; Table 4 and Figure 3A). In contrast, the median OS in the PSM subgroup was significantly longer than that in the NAM subgroup (not reached [95% CI: 18.9–not reached] vs. 8.7 months [95% CI: 1.2–13.4], *p* < 0.001; Figure 3B).

**Table 3:** Patient baseline characteristics between PSM and NAM in nivo/ipi group.

Baseline characteristics		No. of patients (%)		*P*
		PSM	NAM	
		*n* = 16	*n* = 7	
Median age (range)		73 (50–87)	66 (34–76)	0.20
Sex	Female	4 (25)	2 (29)	>0.99
	Male	12 (75)	5 (71)	
ECOG PS	0	12 (75)	6 (86)	>0.99
	1	4 (25)	1 (14)	
	≥2	0	0	
BRAF mutation	Wild-type	15 (94)	7 (100)	>0.99
	Mutation	1 (6)	0	
	Not investigated	0	0	
Stage (AJCC-TNM 8th)	Unresectable stage III	6 (38)	2 (29)	>0.99
	Stage IV (M1a)	2 (13)	1 (14)	
	Stage IV (M1b)	5 (31)	3 (43)	
	Stage IV (M1c)	1 (6)	1 (14)	
	Stage IV (M1d)	2 (13)	0	
LDH level	≤ULN	14 (88)	4 (57)	0.14
	>ULN	2 (13)	3 (43)	
Metastatic organ sites	1	10 (63)	5 (71)	>0.99
	2	3 (19)	1 (14)	
	≥3	3 (19)	1 (14)	
Brain metastasis	Absent	14 (88)	7 (100)	>0.99
	Present	2 (13)	0	
Type of resistance to PD-1 monotherapy	Innate	11 (69)	2 (29)	>0.99
	Acquired	5 (31)	5 (71)	

PSM, palm and sole melanoma; NAM, nail apparatus melanoma; nivo/ipi, nivolumab and ipilimumab; PS, performance status; LDH, lactate dehydrogenase; PD-1, anti-programmed cell death protein 1.

**Table 4:** Overall response between PSM and NAM in nivo/ipi group.

	No. of patients (%)		*P*
	PSM	NAM	
	*n* = 16	*n* = 7	
Complete response	1 (6)	0	
Partial response	3 (19)	0	
Stable disease	4 (25)	1 (14)	
Progressive disease	8 (50)	6 (86)	
Unable to determine	0	0	
Objective response	4 (25)	0	0.27
Disease control rate	8 (50)	1 (14)	0.18

PSM, palm and sole melanoma; NAM, nail apparatus melanoma; nivo/ipi, nivolumab and ipilimumab.

**Figure 4:**
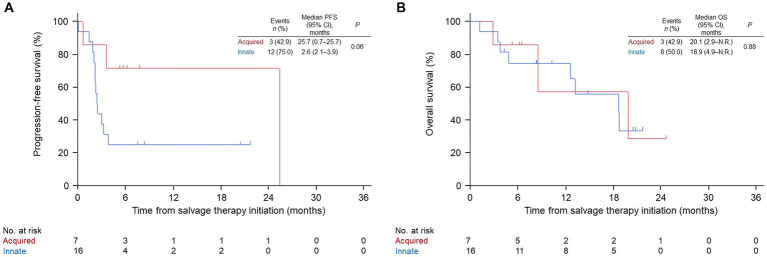
Kaplan–Meier survival curves for innate or acquired resistance to PD-1 monotherapy in the nivolumab and ipilimumab combination group. **(A)** progression-free survival (PFS). **(B)** overall survival (OS). PD-1, anti-programmed cell death protein 1; CI, confidence interval; N.R., not reached.

Patients in the nivo/ipi group were also divided into innate or acquired PD-1 resistance subgroups, which had similar baseline characteristics (Table 5). The ORR and PFS in the acquired resistance subgroup were insignificantly better than those in the innate resistance subgroup (ORR: 43 vs. 6%, *p* = 0.07; median PFS: 25.7 [95% CI: 0.7–25.7] vs. 2.6 months [95% CI: 2.1–3.9], *p* = 0.06; Table 6 and Figure 4A). The subgroups also had similar median OS durations (20.1 [95% CI: 2.9–not reached] vs. 18.9 months [95% CI: 4.9–not reached], *p* = 0.88; Figure 4B).

**Table 5:** Patient baseline characteristics by innate or acquired resistance to PD-1 monotherapy in nivo/ipi group.

Baseline characteristics		No. of patients (%)		*P*
		Innate	Acquired	
		*n* = 16	*n* = 7	
Clinical form	PSM	11 (69)	5 (71)	>0.99
	NAM	5 (31)	2 (29)	
Median age (range)		69 (34–87)	71 (59–78)	0.94
Sex	Female	3 (19)	3 (43)	0.31
	Male	13 (81)	4 (57)	
ECOG PS	0	14 (88)	4 (57)	0.14
	1	2 (13)	3 (43)	
	≥2	0	0	
BRAF mutation	Wild type	15 (94)	7 (100)	>0.99
	Mutation	1 (6)	0	
	Not investigated	0	0	
Stage (AJCC-TNM 8th)	Unresectable stage III	5 (31)	3 (43)	>0.99
	Stage IV (M1a)	2 (13)	1 (14)	
	Stage IV (M1b)	5 (31)	3 (43)	
	Stage IV (M1c)	2 (13)	0	
	Stage IV (M1d)	2 (13)	0	
LDH level	≤ULN	12 (75)	6 (86)	>0.99
	>ULN	4 (25)	1 (14)	
Metastatic organ sites	1	9 (56)	6 (86)	0.54
	2	3 (19)	1 (14)	
	≥3	4 (25)	0	
Brain metastasis	Absent	14 (88)	7 (100)	>0.99
	Present	2 (13)	0	

PD-1, anti-programmed cell death protein 1; nivo/ipi, nivolumab and ipilimumab; PSM, palm and sole melanoma; NAM, nail apparatus melanoma; PS, performance status; LDH, lactate dehydrogenase.

**Table 6:** Overall response by innate or acquired resistance to PD-1 monotherapy in nivo/ipi group.

	No. of patients (%)		*P*
	Innate	Acquired	
	*n* = 16	*n* = 7	
Complete response	0	1 (14)	
Partial response	1 (6)	2 (29)	
Stable disease	3 (19)	2 (29)	
Progressive disease	12 (75)	2 (29)	
Unable to determine	0	0	
Objective response	1 (6)	3 (43)	0.07
Disease control rate	4 (25)	5 (71)	0.07

PD-1, anti-programmed cell death protein 1; nivo/ipi, nivolumab and ipilimumab.

### 3.5. Cox multivariate analysis for PFS and OS

The BRAFi/MEKi group was excluded from the analysis because it was too small. A larger number of metastatic organs (hazard ratio [HR], 6.04 [95% CI: 1.94–18.75] and 7.05 [95% CI: 1.87–26.58]; *p* = 0.002 and 0.004) and NAM (HR, 2.79 [95% CI: 1.35–5.80] and 3.89 [95% CI: 1.72–8.80]; *p* = 0.006 and 0.001) were negatively associated with PFS and OS, respectively. ECOG-PS scores of 1 (HR, 4.67 [95% CI: 1.70–12.81]; *p* = 0.003) and ≥ 2 (HR, 6.31 [95% CI: 1.43–27.83]; *p* = 0.01) were also negatively associated with OS, while a higher AJCC stage was positively associated (stage IV-M1d: HR, 0.03 [95% CI: <0.01–0.36]; *p* = 0.005). PD-1 resistance and salvage therapy types did not affect PFS (acquired resistance: HR, 0.62 [95% CI: 0.30–1.26], *p* = 0.18; ipi: HR, 1.61 [95% CI: 0.65–4.03], *p* = 0.30; nivo/ipi: HR, 1.04 [95% CI: 0.38–2.79], *p* = 0.95) or OS (acquired resistance: HR, 0.49 [95% CI: 0.22–1.11], *p* = 0.09; ipi: HR, 1.42 [95% CI: 0.54–3.77], *p* = 0.48; nivo/ipi: HR, 0.79 [95% CI: 0.26–2.46], *p* = 0.69; Table 7).

**Table 7:** The Cox multivariate proportional-hazards model for progression-free survival and overall survival.

	Progression-free survival				Overall survival		
	Hazard ratio	95% CI	*P*		Hazard ratio	95% CI	*P*
Age at PD-1 monotherapy initiation	1.02	0.98–1.05	0.40		1.03	0.99–1.07	0.21
Male sex	0.53	0.25–1.12	0.10		1.23	0.51–2.95	0.64
ECOG PS							
0	Reference						
1	1.81	0.77–4.28	0.18		4.67	1.70–12.81	0.003
≥2	2.37	0.63–8.89	0.20		6.31	1.43–27.83	0.01
AJCC-TNM 8th stage							
Unresectable stage III	Reference						
Stage IV (M1a)	2.08	0.81–5.34	0.13		2.17	0.76–6.21	0.15
Stage IV (M1b)	0.63	0.22–1.82	0.39		1.10	0.32–3.80	0.89
Stage IV (M1c)	0.39	0.12–1.28	0.13		0.54	0.11–2.60	0.44
Stage IV (M1d)	0.73	0.14–3.91	0.68		0.03	<0.01–0.36	0.005
Elevated LDH	1.12	0.48–2.64	0.79		0.64	0.23–1.82	0.41
No. of metastatic organ							
1 organ site	Reference						
2 organ sites	1.34	0.53–3.38	0.54		0.83	0.30–2.32	0.73
≥3 organ sites	6.04	1.94–18.75	0.002		7.05	1.87–26.58	0.004
NAM clinical form	2.79	1.35–5.80	0.006		3.89	1.72–8.80	0.001
Acquired resistance type	0.62	0.30–1.26	0.18		0.49	0.22–1.11	0.09
Salvage therapy							
Chemotherapy	Reference						
ipi	1.61	0.65–4.03	0.30		1.42	0.54–3.77	0.48
nivo/ipi	1.04	0.38–2.79	0.95		0.79	0.26–2.46	0.69

PD-1, anti-programmed cell death protein 1; PS, performance status; LDH, lactate dehydrogenase; NAM, nail apparatus melanoma; ipi, ipilimumab; nivo/ipi, nivolumab and ipilimumab.

## 4. Discussion

This study investigated the therapeutic efficacy of salvage therapy after PD-1 MF in patients with advanced AM. The Kaplan–Meier analysis suggested that nivo/ipi therapy resulted in better PFS and OS than the other salvage therapies; however, the difference was not significant. NAM was associated with significantly longer OS than was PSM in the nivo/ipi group. Cox multivariate analysis showed that salvage therapy selection and type of resistance to PD-1 did not affect the PFS or OS. Only a larger number of metastatic organ sites and NAM were negatively associated with PFS and OS.

Several studies have reported the efficacy of salvage therapies after PD-1 MF in patients with advanced melanoma. Wang et al. retrospectively investigated the clinical efficacy of nab-paclitaxel or temozolomide in combination with antiangiogenic drugs, including endostatin or apatinib, as salvage therapies after PD-1 MF in 69 Chinese patients with advanced melanoma (30). The ORR and DCR were 5.8 and 63.8%, respectively, and the median PFS was 3.0 months. Although that study included 23 patients with AM (33.3%), the detailed efficacy in this group was unavailable. A previous study among 355 patients with advanced melanoma resistant to PD-1 or PD-L1 monotherapy demonstrated that ipi plus anti-PD-1 as a salvage therapy showed better clinical efficacy than ipi alone, with a higher ORR (31.1 vs. 13.0%, *p* < 0.001) and longer median PFS (3.0 vs. 2.6 months, *p* = 0.002) and OS (20.4 vs. 8.8 months, *p* < 0.001) (31). Arance et al. investigated the clinical efficacy of lenvatinib (multikinase inhibitor) plus pembrolizumab in 103 patients with advanced melanoma resistant to PD-1 or PD-L1. ORR, median PFS, and median OS were 21.4%, 4.2 months, and 14.0 months, respectively (32). Both studies, conducted in Australia, Europe, Canada, and the USA, did not report the proportions of the different clinical forms of melanoma (NCAM, AM, mucosal melanoma, among others) that were enrolled. Few patients with AM may have been included in those studies (31, 32) because of the rarity of AM in the Caucasian population. As the first-line PD-1 efficacy in patients with AM has been reported to be lower than that in patients with NACM (20), the clinical melanoma form could influence salvage therapy efficacy after PD-1 MF. Regarding AM, Bhave et al. reported that the ORRs in the second line ipi plus PD-1 and ipi alone groups after PD-1 monotherapy were 24 and 14%, respectively (19). Meanwhile, the PFS and OS in each group were not investigated. In that study, most patients with AM were Caucasian (75%). In Asian patients with AM, the efficacy of salvage therapy remains unclear. To our knowledge, this was the largest study to investigate the clinical efficacy of salvage therapy after PD-1 MF in a homogenous population (Japanese patients alone) with advanced AM. Unlike the previous study (31), this study suggested that nivo/ipi was not more effective than the other salvage therapies after PD-1 MF in patients with advanced AM.

In this study, the efficacy of nivo/ipi in PSM was superior to that in NAM. Cox multivariate analysis also indicated a significantly poorer PFS and OS in the NAM group than in the PSM group. These data imply that the prognosis after PD-1 MF depends on the clinical form of AM (PSM or NAM) rather than the choice of salvage therapy. A recent retrospective study compared first-line nivo/ipi and PD-1 therapies in 254 Japanese patients with advanced AM (22). The study showed that nivo/ipi led to a higher ORR than PD-1 in the NAM group (61.5 vs. 9.8%, *p* < 0.001), although a similar ORR was noted in the PSM group (31.3 vs. 18.9%, *p* = 0.44). Cox multivariate analysis also demonstrated that nivo/ipi was an independent predictor of prolonged PFS in the NAM group (HR, 0.23, *p* = 0.002) (22). Based on these data, nivo/ipi should be selected as the first-line treatment rather than salvage therapy after PD-1 MF in patients with NAM.

The correlation between PD-1 monotherapy resistance type and the salvage therapy efficacy after PD-1 MF is not fully investigated. A previous study reported that the efficacy of ipi plus PD-1 was superior to that of ipi alone in both the acquired and innate groups; however, the differences were not significant (31). This study demonstrated that the acquired resistance subgroup showed a trend toward higher ORR and PFS in the nivo/ipi group, though not significant. Furthermore, Cox multivariate analysis detected no significant predictors for either PFS or OS. The efficacy of salvage therapies may not depend on the resistance type of PD-1 monotherapy.

This study had some limitations. The study design was retrospective, with a slightly uneven distribution of patient characteristics between the treatment groups. Additionally, patient characteristics were assessed at PD-1 initiation, while those at salvage therapy initiation were unavailable. PD-1 involves two drugs (nivolumab and pembrolizumab) at different doses and treatment intervals. The baseline *KIT* and *NRAS* mutation status was unknown because the Japanese health insurance does not cover routine molecular testing for these mutations. Although *KIT* mutations were detected in approximately 40% of Japanese patients with AM (33), KIT inhibitors, such as imatinib and sunitinib, are not approved for treating advanced melanoma in Japan. Therefore, KIT inhibitors were not evaluated as salvage therapies in this study. The analysis did not include programmed death ligand 1 expression status in tumor cells at baseline, which might be a confounding factor. Finally, the sample was small, particularly in the BRAFi/MEKi and cytotoxic chemotherapy groups, and the follow-up was relatively short.

In conclusion, nivo/ipi was associated with numerically higher median PFS and OS than were the other salvage therapies, with insignificantly better PFS and OS after PD-1 failure in patients with AM. In subgroup analysis of patients treated with nivo/ipi, OS was significantly longer in the PSM subgroup than in the NAM subgroup. Salvage therapies, including nivo/ipi, showed low clinical efficacy in patients with NAM; therefore, nivo/ipi should be used as the first-line therapy for NAM. Further prospective clinical trials focusing on AM and including larger samples and longer follow-up periods are required to determine accurate differences in clinical efficacy between salvage nivo/ipi and other salvage therapies. Moreover, more effective salvage therapies must be developed in the future.

## Data availability statement

The raw data supporting the conclusions of this article will be made available by the authors, without undue reservation.

## Ethics statement

The studies involving humans were approved by Ethics Committee of Saitama Medical University International Medical Center. The studies were conducted in accordance with the local legislation and institutional requirements. The ethics committee/institutional review board waived the requirement of written informed consent for participation from the participants or the participants’ legal guardians/next of kin because this study is a retrospective study.

## Author contributions

TMo: formal analysis, writing—original draft, and writing—review and editing. KeN, NY, YKi, OY, SY, TIn, HK, YNakai, SF, TT, TMae, SM, AO, MN, NB, TIs, SS, NF, RT, TK, YKu, TMat, KoN, MO, and TO: investigation and writing—review and editing. YNakamura: supervision, conceptualization, methodology, project administration, funding acquisition, investigation, and writing—review and editing. All authors contributed to the article and approved the submitted version.

## Funding

This work was supported by the Japan Agency for Medical Research and Development (grant numbers JP21ck0106508h0003, 22ck0106765h0001, and 23ck0106765h0002) and the National Cancer Center Research and Development Fund (grant numbers 2020-J-3 and 2023-J-3). The institutions and funding sources were not involved in the study design; collection, analysis, and interpretation of data; writing of the report; or decision to submit the article for publication.

## Conflict of interest

TMo and SS have received honoraria from Ono Pharma. KeN has served as a consultant and/or received honoraria from Novartis, Bristol-Myers Squibb (BMS), Merck Sharp & Dohme (MSD), Novartis, and Ono Pharma. NY has received research funding from BMS, MSD, Novartis, Ono Pharma, and Takara Bio, and has served as a consultant and/or received honoraria from BMS, MSD, Novartis, and Ono Pharma. YKi, SY, HK, and NF have received honoraria from Novartis and Ono Pharma. OY has received research funding and/or honoraria from Ono Pharma. TIn has received honoraria from BMS, MSD, and Ono Pharma. SF has received research funding from Ono Pharma and honoraria from BMS, MSD, and Novartis. TT, TMae, and SM have received honoraria from BMS, MSD, Novartis, and Ono Pharma. AO has served as a consultant and/or received honoraria from BMS, MSD, Novartis, and Ono Pharma, and has received research funding from Eisai. TIs has served as a consultant and/or received honoraria from Ono Pharma, Pfizer, BMS, and Novartis. YNakamura has served as a consultant and/or received honoraria from BMS, MSD, Maruho, Ono Pharma, Sanofi, Sun Pharma, Tanabe Mitsubishi Pharma, and Torii.

The remaining authors declare that the research was conducted in the absence of any commercial or financial relationships that could be construed as a potential conflict of interest.

## Publisher’s note

All claims expressed in this article are solely those of the authors and do not necessarily represent those of their affiliated organizations, or those of the publisher, the editors and the reviewers. Any product that may be evaluated in this article, or claim that may be made by its manufacturer, is not guaranteed or endorsed by the publisher.
